# On Epilepsy
*Epilepsy and the Allied Affections. By Charles Bland Radcliffe, M.D. London: John Churchill.Du Prognostic et du Traitement Curatif de l'Epilepsie. Par Th. Herpin. Paris: Baillière.


**Published:** 1855-01-01

**Authors:** 


### Art. III.—ON EPILEPSY.*

The most accurate exponent of the advance of science is ever to be sought in the extent to which it is applicable to the true interests of man; in the extent to which it promotes *wisdom*, as distinguished from mere *knowledge*, and in the amount of its applicability rather to the wants of man, than to the gratification of his curiosity. In the infancy of any *science*, facts are imperfectly observed, loosely described, and their significance misunderstood; they appear as a chaos of phenomena, unconnected, or but very feebly connected, by hypothesis; and it is only when their true bearing and mutual connexion and dependance being clearly perceived, they become constantly recurring illustrations of one grand principle, that the parent *science* becomes worthy of its name. Casual observation, wonder, hypothesis, mystery, over-appreciation, and neglect, are a few only of the preliminary phases through which truth has to pass, before obtaining its proper recognition, and paying its proper quota to the service of mankind.

These remarks are suggested by a perusal of the works before us, where we find the three sciences of Physiology, Electricity, and Statistics, applied to the elucidation of the phenomena of a disease, which,

* Epilepsy and the Allied Affections. By Charles Bland Radcliffe, M.D. London: John Churchill.

Du Prognostic et du Traitement Curatif de l'Epilepsie. Par Th. Herpin. Paris: Baillière.

if not one of the most fearful scourges of *the race*, is certainly one of the most formidable and incomprehensible which can attack the individual, whether viewed in reference to its immediate invasion, or its ulterior consequences.

These branches of knowledge have not been exempt from the various phases of opinion already alluded to. When Harvey discovered the circulation of the blood, it appeared to those of sanguine and hopeful temperament that now a sure and certain method of curing all manner of disease was, or would speedily be, indicated. This was the stage of over-appreciation; it passed by a natural transition, through disappointment, to neglect. And so has it been with regard to physiological discoveries in general, till the science, instead of serving as the true foundation for distinguishing and treating disease, has but too frequently been prostituted to the co-ordinating of theories, or the justification of a foregone conclusion. Electricity was long the plaything of the child, the toy of the philosopher; it is now the potent analyser of mysterious compounds, the vehicle of a nation's thought; and combined with physiological reasoning, we find it in the hands of Dr. Radcliffe, applied as a powerful calculus to the hitherto crude and incongruous mass of facts and opinions, bearing upon muscular action in general, and epileptic convulsions in particular. Statistics have been alternately the weapon, the jest, and the shield of the statesman; yet, carefully and properly applied to the investigation of this disease by M. Herpin, we shall find it lead to many useful and interesting results, as regards its prognosis and treatment.

Taking these two works as our text, and availing ourselves of other sources of information, where it may appear necessary, we shall proceed to examine what is the present state of our knowledge, and what are our future prospects in reference to this interesting and fearful disease.

For facilitating this investigation, we propose to ourselves the following subjects of inquiry:—

1. What are the phenomena of epilepsy?
2. What are its varieties?
3. What is its general pathology?
4. What are the conditions favouring the development of the epileptic tendency?
5. What are the influences presiding over periodicity?
6. What place in the natural history of disease can we assign to epilepsy?
7. How is epilepsy distinguished from other diseases?
8. What is our prognosis, generally, and in any individual case?
9. What is the proper and rational treatment of epilepsy?

I. *What are the phenomena of Epilepsy?*—There are two distinct forms in which the *fit* of epilepsy appears—the epileptic convulsion, and the epileptic vertigo; the *grand mal* and the *petit mal* of the French writers. The general characters are—loss or great diminution of consciousness, generally with convulsion, but occasionally with extreme relaxation, always with great modification of the muscular system; oppression and embarrassment of the respiratory and circulatory functions; the attack lasting from a few seconds to many hours, terminating very frequently, if not *usually*, in a state of apparent health; recurring sometimes not at all, but most frequently at intervals, not usually marked by any regularity, though this is subject to exceptions. We extract Dr. Radcliffe's vivid portrait of the epileptic convulsion entire:—

"The fit is ushered in by a cry or scream, and the patient is at once dashed to the ground. The whole frame is seized with violent and frightful convulsions, the features are horribly drawn, the head is twisted to one side, the eyes are distorted and half protruded from their sockets, the teeth are gnashed together, and the tongue is mangled between them until the mouth overflows with bloody foam, the limbs are dashed about violently, the chest is so fixed that all proper respiration is at an end, and, last of all, the bladder, intestines, and seminal vesicles participate in the spasm and expel their contents. The temperature of the skin is usually below the natural standard, and the hands and feet are cool or actually cold; but, in the course of the paroxysm, and as the asphyxial symptoms gain ground, the head and neck become warm and tumid, the tumidity rapidly increases, and the colour changes from dull red to deep blue or black. In a less degree this change extends to the rest of the body, but, as a general rule, the hands and feet remain cool and pale throughout, or only acquire a slight venous or bluish tinge. The pulse rapidly becomes insensible, or nearly so, though the heart beats with tumultuous violence. There is no consciousness whatever, and the most violent stimulants fail to rouse the dormant senses. For some time after the violence of the fit is over, the limbs are shaken by passing quivers, and the breathing interrupted by sobs or gasps, but at length these residuary troubles end in a state of comatose sleep, in which the breathing is often loud and stertorous. Then the lungs resume their natural action, and, consequent upon this change, the veins of the head and neck become unloaded, the colour and pulse return, and the patient wakens to an obscure and troubled consciousness."—*Epilepsy and Allied Diseases*, pp. 49—51.

The less formidable attack, in appearance at least, is without convulsion, turgescence of face, or foaming of the mouth. There is sudden loss, or great diminution and embarrassment of the consciousness, relaxation of the muscular system, tottering, staggering, or falling; a cold clammy skin, a feeble pulse, and, in many cases, an almost immediate return of the faculties. Still milder forms than this are described, and, indeed, in the confirmed epileptic we meet with every variety of attack, from the simple vertigo, which lasts but an almost inappreciable moment, to the violent and long-continued convulsion above described. Some patients are only affected by the vertigo, and never have the convulsion; yet we cannot consider their cases as less serious than the others, for we have the high authority of M. Foville for asserting that intellectual degradation occurs more constantly and more quickly amongst those affected by vertigo, or *petit mal*, than amongst those who have *only* the convulsions, or *grand mal*. Most frequently, however, the forms are found combined in the same individual. In sixty-eight cases mentioned by M. Herpin, there were only five where vertigo existed alone.

Most frequently these attacks, whether of vertigo or convulsion, take place without warning. In a few instances there are distinct premonitory signs, which may be taken advantage of by the sufferer. Thus, Dr. Radcliffe observes, that, "on the eve of a fit, confirmed epileptics are noticed to sit or move about in a moping and listless manner;" to complain of chills and shiverings, or of faintness and sickness. "The respiration is interrupted by frequent sighs; the pulse is weak, irregular, and slow." Occasionally there is headache, dazzling of the eyes, singing in the ears, and other excitements of sensation; slight flushing of the face, dilatation of the pupils, and extreme irritability of temper. In some rare instances, there is, immediately before, or at the commencement of, the attack, a phenomenon of a more specific nature. For the following description we are indebted to M. Foville. "A peculiar sensation, it may be of cold, pain, heat, or itching, is developed suddenly in a toe, a finger, a limb, in the belly or the back, and from the point whence it originates, mounts gradually to the head; it arrives there, and immediately the patient falls (as if struck); the convulsions break forth at once." This sensation has received, from the earliest times, the name of *aura epileptica*. It is rare; so much so, that by many its existence is doubted or ignored, and by others, explained in a different manner. Thus, M. Herpin considers it as nothing more than the commencement of the tonic spasm of the muscles of the limb. This view can scarcely be admitted; we know that modifications of sensation do frequently precede an attack; and in an affection where sensibility and motility are equally affected, it seems but reasonable to suppose that the attack may be heralded sometimes by changes in the one class of nerves, and sometimes in the other. For an interesting *resumé* of the various phenomena of a sensor, motor, or psychical character, which occasionally precede the attack of epilepsy, we refer our readers to Romberg's treatise on

" Diseases of the Nervous System," article—Epilepsy, and to the article —Epilepsie, in the " Dict. des Sciences Medicales," by M. Esquirol.

Of the frequency of the occurrence of premonitory signs in general, very different accounts are given by various authors. Dr. Radcliffe considers them nearly constantly to be observed; Professor Romberg notices them in about one half of his patients; M. Herpin states the proportion to be about one-fourth; M. Georget states that not more than four or five per cent. of those attacked with an epileptic seizure have any premonition; M. Beau gives the proportion of seventeen per cent.; M. Foville, M. Esquirol, and Dr. Cheyne give no numerical ratio, but state that in much the greater number of cases there are no precursory symptoms. We believe, however, that careful observation would most frequently detect some changes in the system, analogous to those above described.

Though there be this difference of opinion concerning the outset of the attack, there is but little doubt as to the results. Except in the very slightest seizures, and in the epileptic vertigo, the fit always leaves behind it some sequelæ, such as headache, drowsiness, pain in the limbs, stiffness and soreness of the whole body, pain in the back of the neck, swollen and bitten tongue, ecchymoses, and bruises. These all appear to be the natural results of the attack, produced chiefly mechanically. But there are other effects, more serious in character, and more insidious in their invasion. Death but rarely occurs in the fit; after a day or two, however severe the attack, the patient appears in his usual health; but, by-and-by, another and another fit supervenes, and the nervous centres begin to suffer, and not to recover their due functions in the intervals. The features alter and become ugly (Esquirol); the limbs become gradually emancipated from the control of the will; hemiplegia often occurs; the memory becomes feeble; and we observe in the intervals a diminution of the intelligence, which, gradually augmented, brings on at length a state of confirmed dementia. These fearful results have been known to occur after one fit (Esquirol) in children, but this is not usual. On the other hand, we have known many epileptics whose intellect has not appeared to suffer in the least by attacks, severe, long-continued, and of many years' duration. Dr. Cheyne gives similar instances, (article—Epilepsy; " Cyclopædia of Practical Medicine.") Yet we may take it for granted as a general rule, that such severe functional derangements, even if in the beginning they be no more than functional, cannot continue long without leading to serious organic mischief, and deterioration of the mental faculties in the great majority of cases.

A few words on some of the individual symptoms will conclude our remarks on the phenomena of epilepsy.

The premonitory symptoms are evidently due to modifications of innervation, and of the circulation in the nervous centres, or to disorder of the particular organ or viscus in which the exciting cause of the convulsion is situated. The "aura" may sometimes be the commencement of spasm, but more frequently we believe it to be indicative of a change in the nervous centres themselves, and to be strictly a reflected sensation, a centrical impression. The scream with which the attack is ushered in is one of the most fearful sounds in nature. Many accounts are given, some ludicrous, and some very melancholy, of the effects produced upon excitable persons hearing it,—its nature is not well understood. That it is not indicative of pain or fear, at least in all instances, is capable of clear demonstration. We are well acquainted with an epileptic patient who screams dreadfully on the attack, and who has frequently described to us the sensation of the invasion as *most delightful*, and this though dreading the attack to the utmost extent. He says that he hears sounds and sees colours all of the most beautiful character, but cannot clearly satisfy himself at the time *which is sound and which is colour*,—

"The hues seemed music, and the music, hues."

He has no sensation of pain whatever. All writers concur in affording illustration of the same principle. The noise is most probably produced by the first convulsive action of the chest, together with that of the larynx.

The convulsion is partly *tonic*, though chiefly *clonic*, (the existence of the former may often be traced even during the most violent prevalence of the latter;) it may be general, more frequently it is partial; it may be wanting altogether, as in the vertiginous form, and many varieties of the *petit mal*. The *fall* generally precedes the convulsion, but in some instances follows it, as in a case cited by Esquirol. The embarrassment of the respiratory function is, we believe, correctly attributed by Dr. Radcliffe to the spasmodic fixture of the parietes of the chest; but at the same time it appears that changes take place in the organs themselves, as evidenced by the increased secretion of mucus in the trachea. The loss of sensibility appears to be simultaneous in its invasion with the convulsion and fall. It is generally complete, but not invariably.

It is a strange and suggestive fact, that whilst those diseases which are obscure and variable in their symptoms, proteiform in their manifestations, insidious in their invasion, and of difficult diagnosis, have been discovered, hunted to their homes, and traced to their proximate cause—epilepsy, which has not varied in its phenomena since the days of Hippocrates, which is easy of recognition, plain and palpable in its attack and its results, still remains one of the *opprobria medicinæ*. It is interesting, as an illustration of the constancy of this disease, to compare the account given by the great father of medicine with that which we have given above. He says—

"The patient loses his speech (and intellect), and chokes, and foam issues by the mouth; the teeth are fixed, the hands are contracted, the eyes distorted; he becomes insensible, and in some cases the bowels are evacuated. He kicks with his feet . . . . and these symptoms occur sometimes on the left side, sometimes on the right, and sometimes on both." Aretæus and Paulus Ægineta give similar or identical accounts.

Such, constant and well marked, have been the symptoms of this disease since the days of Hippocrates; and yet it would appear that no step has been taken in the meantime, tending to the discovery of its real cause and essential nature. But nature cannot be ever obdurate to the patient observer of her phenomena, and we hope to indicate shortly, that an advance is being made in the right direction.

II. *The Varieties of Epilepsy.*—A very natural division of the subject has always suggested itself to systematic writers on this disease—viz., into E. Cerebralis and E. Sympathetica, according as the root of the disease was supposed to be in the brain or in some distant organ. We prefer the terms E. Centrica, and E. Excentrica, the division being essentially the same, but the expression more comprehensive, as including in the former not only the brain, but the spinal cord. The second grand division has again been subdivided into various classes, taking their names from the special organ supposed to be affected, as E. Stomachica, E. Hepatica, E. Nervosa, E. Uterina, E. a Dolore (Dr. Cheyne). We venture, however, to suggest that, in a nosological point of view, these divisions are unnecessary and uninteresting; though, as affecting the treatment, their recognition is important; but, considered as a disease simply, the manifestations are alike in all these cases, and, therefore, not requiring separate description. The preliminary symptoms, however, will sometimes differ, obviously in accordance with the derangement of these special functions, and this will be of essential service in the treatment.

III. *The Pathology of Epilepsy.*—The most cursory view of the subject leads us at once to the nervous centres as the source of, or agent in, the production of these strange phenomena; but having arrived there, we seem as far from the truth, practically, as ever. Is it a disease of nervous excitement? Why, then, is consciousness destroyed or suspended?—Is it one of depression? Why, then, is muscular action so violently increased?—What is the condition of the brain on the eve of, and during an attack of epilepsy? Is it congestion? Why, then, do the symptoms *decrease* when the congestion is on the increase towards the close of the fit?—Is it inflammation? This is obviously incredible, from the very transient nature of the attack. These are important questions, and deserve the most serious consideration. An answer to them is found in Dr. Radcliffe's work, marked by such originality of thought, and such earnest research into the phenomena, that we cannot resist laying it, at some length, before our readers. And in order to do this, it will be necessary to enter into our author's views on the subject of muscular contraction in general, as, without this, his pathology of epilepsy would not be comprehensible.

At p. 41 we find the following law stated, which contains a most remarkable deviation from the received views of muscular motion, but which is the basis of Dr. Radcliffe's account of the pathology of epilepsy and all allied convulsive affections:—

> "ALL STIMULANTS, VITAL AND PHYSICAL, ANTAGONIZE MUSCULAR CONTRACTION, AND CONTRACTION HAPPENS FROM ORDINARY MOLECULAR ATTRACTION, WHEN THE MUSCLE IS NOT STIMULATED."

This opinion our author founds upon a great number of facts and experiments, of which the following is an abstract:—

1. Rigor mortis (analogous to ordinary muscular contraction) occurs after all stimulus has ceased. It may be proper to mention that "stimulus" includes the sum of the influences brought to bear upon muscle, such as innervation, blood, temperature, and the like. Rigor mortis, then, only occurs on the cessation of "stimulus."

2. The dactos contracts on the application of cold, which is but the abstraction of the stimulus of heat; the skin under the same circumstances shrivels.

3. "Comparing voluntary and involuntary muscles, their contractibility is found to be related, in an inverse ratio, to the supply of nerves (p. 7), and to the supply of blood (p. 8)," and convulsion occurs on bleeding an animal to death at the shambles. Also rigor mortis may be relaxed by the injection of warm blood into the vessels.

4. The argument adduced from mechanical irritation as inducing contraction, and from the action of the hollow viscera, as the uterus and bladder upon their contents, does not admit of condensation. We must refer our readers to the work itself, pp. 8 to 11.

5. The testimony which electrical phenomena bear to this view are very closely investigated and clearly stated. The result of them is, that an electrical current exists in a muscle *during rest*, and ceases altogether during contraction, the needle of the galvanometer at such times pointing to zero, as it does also in cadaveric rigidity. It also appears from these experiments, that artificial electric currents produce contraction in a limb, by *neutralizing* the already-existing natural current.

6. From the action of cold and heat upon the animal tissues, it appears that the former always produces contraction, and the latter relaxation.

7. The condition of the bloodvessels, under various circumstances, affords, according to our author's view, further corroboration of the law. Thus, "joy flushes the skin, and fear blanches it; in other words, the superficial capillaries expand when the nervous energy is exuberant, and shrink when it is deficient." (p. 25.) In inflammation and various pathological states of the system, there are other illustrations of the same principle. This question is still more fully discussed in a previous work by the same author, on "Vital Motion."

8. It is impossible to condense the argument deduced from the action of the heart, so as at once to make it comprehensible, and bring it within our limits. We can but state the result arrived at, viz., that the diastole of the ventricle is the *active state*, and is synchronous with the greatest innervation, and the most free supply of blood to the vessels of the heart; that the contraction is a passive state, synchronous with the diminution of innervation, and consequent upon that and the diminished supply of blood. This our author supposes also to furnish a solution of the mystery of the rhythmical action of the heart; but for the full illustration of this part of the subject, we can but refer to Chapter 3, which contains many interesting and suggestive remarks, and which concludes thus:—

"The doctrine, then, that all stimulants, vital and physical, antagonize muscular contraction, and that contraction happens from ordinary molecular attraction when the muscle is not stimulated, may be said to receive its final physiological confirmation in the physical explanation which it affords to the three great and fundamental problems in physiology,—muscular contraction, the movements of the blood in vessels independently of the heart, and the rhythm of the heart. And hence the necessity for the full investigation of the law of muscular contraction, before entering upon the investigation of epilepsy, and other disorders, in which muscular contraction is in excess; for if the old doctrine that muscular contraction is the result of stimulation must fall to the ground, then all pathological deductions founded upon that doctrine must fall along with it."

To complete the physiological view of this question, it is incumbent upon us to allude to those phenomena which appear to militate against this view, or which at least require further elucidation, before they can be deemed illustrations of the same general law.

1. The phenomena of muscular contraction differ in many respects from molecular attraction,—in its sudden occurrence, in the absence, or almost absence, of diminution in the absolute bulk of the muscle,—in its great lessening of length, and great increase in breadth and thickness.

2. In diseases of deficient innervation and circulation, as in chlorotic, anæmic, and syncopoid states, muscular contractility and tonicity are low, and only as exceptions become spasmodic.

3. In cases where, from injury or disease, the nervous energy is abstracted, as in paralysis, or the division of a nerve, the *rule* is, muscular relaxation.

4. The phenomena of rigor mortis do not occur at once, sometimes not for hours after the cessation of life, and the consequent abstraction of stimulus.

5. It appears from general testimony that convulsion may occur from plethora, as well as from anæmia, as Esquirol observes, that it is in accordance with many facts, that Hippocrates and all subsequent observers have regarded plethora as one of the causes of epilepsy.

These and similar facts may serve to indicate the class of phenomena which do not appear subservient to the same law. We do not doubt, however, that so acute a physiologist as Dr. Radcliffe has foreseen and provided against these apparent objections,—indeed, some of them are urged by himself; but until further explanation of them is afforded, we must allow the question to remain *sub judice*,—a more full discussion of the subject would lead us too far from our purpose at present.

In the meantime, adopting these physiological views, we are now prepared to understand our author's pathological opinions on the nature of epilepsy and convulsion in general. Commencing the investigation by interrogating the three great systems, the vascular, the nervous, and the muscular, he finds that in each there is a depression of proper power, the circulation low, the system "unnerved," and the muscular system indicating want of tone and energy, all which is clearly demonstrated.

"Viewed in this manner, the vascular and nervous systems of the epileptic, as well as the mobile structures in which the convulsive phenomena are manifested, are seen to present unequivocal evidences of inactivity; and this inactivity—so far, at least, as the vascular and nervous systems are concerned—is found to be most marked in the fit itself."

"It is, then, sufficiently evident that epilepsy cannot be caused by any excitement of the muscles, consequent upon the excessive supply of nervous or any other stimulus. On the contrary, everything is in harmony with the physiological premises, and, as might be anticipated from these premises, the convulsion would seem to depend upon *want* of vital stimulation, which want had allowed the molecular attraction of the muscles to come into play, and gain the ascendancy."—*Epilepsy*, pp. 59—61.

In that part of the work which is devoted to the affections allied to epilepsy, and marked by convulsion, tremor, or spasm, we find still ampler confirmation of these views; but as it is our intention to confine our remarks chiefly to epilepsy itself, we must leave these for some future occasion. Having, then, got a clear and definite statement of the general pathological condition of the system, we are prepared to enter upon our next question:—

IV. *The Conditions favourable or conducive to the Development of the Epileptic tendency.*—In answering this question, we shall take advantage of M. Herpin's division of the subject, and examine successively,—

1. Hereditary tendencies.
2. Anatomical conditions.
3. Physiological conditions.
4. Hygienic conditions.
5. Morbid antecedents.

1. It is generally acknowledged that the tendency to epilepsy is hereditary, not *always* in the direct line of ancestry, but either so, or in collateral branches; thus Boerhaave observes:—" *Silente sæpe morbo in genitore, dum ex avo derivatur in nepotem.*" General as this admission is, the statistics are rare by which its absolute frequency could be determined. M. Herpin gives us the particulars of 68 cases, with all the information which could be gathered as to the family affections. The result is interesting, not only as showing absolutely that this class of affections is hereditary, but as indicating those diseases which seem most closely allied to it. Thus he found 11 cases of epilepsy, 24 of mental alienation, 11 of apoplexy with hemiplegia, 13 of chronic meningitis and hydrocephalus, 2 of general paralysis, besides a few isolated instances of suicide, melancholia, &c., and 1 of softening of the brain. Some of these affections were found in more members than one of the same family, so that part of the 68 cases might appear free from the hereditary tendency; but it must be remembered that there are very great difficulties in the way of ascertaining these facts, and that it is more than probable that, could everything relating to the antecedents of an epileptic be known, the instances where the disease appears unpreceded by any of these, its allies, would be very rare. Dr. Cheyne, indeed, considers that it never originates in a family except by exaltation of the strumous diathesis, through intermarriage, or some accidental cause. To this we shall have to refer again.

2. The anatomical conditions which appear to favour the development of epilepsy are various and doubtful, and from their frequent absence and want of constancy, throw but little light upon the nature of the disease. In an epileptic who has had but few attacks, whose intellects or muscular powers have not permanently suffered, and who has died from accident or from some other disease, a *post-mortem* investigation will probably reveal no lesion whatever of the nervous centres, or, as M. Foville observes, "We may, perchance, meet with a tubercle, a cancer, an osteo-calcareous production, which may be regarded as the occasional cause of the disorder; but the disorder has disappeared, *the tubercle still remaining, and no symptom betraying its presence.*" According to the investigations of the Wenzels, the most frequent alteration is found in the pineal body, and they supposed this to be always the case in centric epilepsy. In those who die during an attack, the most constant appearances are those of congestion and extreme gorging of the vessels, but this, as Dr. Radcliffe observes, is evidently due to the action of the fit and to the manner of death. In old, confirmed cases, besides these appearances, we find marks as of the effects of long-continued modifications of the circulation, as induration, or sometimes softening of the white matter, changes in the appearance, also, of the grey substance, and almost always enlargement of the vessels of the brain. Of the special alterations of structure we cannot speak, but must for details refer to systematic works on the subject. Suffice it to say, that all imaginable morbid conditions have been met with, but can scarcely be considered as the causes of the disease, inasmuch as they exist when the disease itself is not actively manifested; and the disease frequently exists with equal or greater virulence when no such changes are to be met with. The same observation applies with still greater force to those anatomical conditions in various organs, which are found in epilepsy originating in irritation at the distal extremity of nerves, in what we have called "excentric epilepsy."

3. *Physiological conditions.* — Amongst these we have, perhaps rather irregularly, included *sex*. It appears, from reports of hospitals, that females are much more frequently affected than males. M. Herpin gives the proportion as 6 to 5; Frank, of 8 to 7. Esquirol mentions, that in the *Salpêtrière* there are 389 women, and at the *Bicêtre* 162 men, in 1813. Georget states, that in 1820, the relative numbers were 324 and 160. *Age* appears to have a material influence in predisposing to epilepsy. From various documents by Leuret and others, it appears that nearly 70 per cent. are attacked before the age of 20. Real congenital epilepsy is very rare, not occurring in more than 1 per cent. One-fourth appear to be attacked before 5 years old; from 5 to 10, not more than 3 per cent. occur; from 10 to 15, and from 15 to 20 years, about one-fifth each. With regard to the after ages, the conclusions appear not sufficiently ascertained. The influence of temperament, of dentition, and of the establishment of menstruation, has yet to be determined. They appear to be small, though this is not in accordance with the popular impression. The *recurrence* of the function of menstruation, however, may frequently be an exciting cause in those otherwise predisposed to the affection. The proportion of married epileptics is very small compared to the unmarried; but this is no etiological indication, as cause and effect here mutually react.

4. *Hygienic conditions.*—We have no accurate means of judging of the proportion of epileptics among the rich and the poor. Hospital practice gives no assistance—private practice is not a correct test; but out of M. Herpin's 68 cases, 21 belonged to rich families, and 26 to workmen in comfortable circumstances. Of the rest, only 11 were in positive indigence. It needs little proof that excess of various kinds —drunkenness, gluttony, and excessive intellectual occupation, having a tendency to the general depression of the powers—tends to favour the epileptic condition. We have no account of moral causes, except as they act as *exciting* causes.

5. The morbid antecedents which have been observed in patients afterwards epileptic are often of a tubercular nature. Besides which we notice mental alienation, hydrocephalus, infantile convulsions, chorea, hysteria, nightmare, and somnambulism.

The exciting or accidental causes are innumerable—strong impressions on the senses, as pain, startling sounds, flashes of lightning; mental emotions, chiefly those of a depressing nature, but sometimes the contrary—fright, grief, extreme fatigue, anger, drunkenness, self-abuse. The excentric epilepsy may be brought on by anything tending to the derangement of its particular seat, as an overloaded stomach, an engorged liver, an irritated uterus, a calculus in the pelvis of the kidney, or the like.

Certain circumstances favour or impede the operation of the accidental cause upon the constitutional tendency. Among these are the season of the year and the time of day. In accordance with Dr. Radcliffe's pathological views, cold seasons seem to be about twice as favourable to the development of the attack, as warm ones. There seems to be a difference of opinion as to the relative frequency of attacks in the day and in the night. Dr. Radcliffe and Leuret consider that the fits happen most frequently by night. M. Beau gives an equal proportion. M. Herpin decides that, though the most violent attacks occur by night, the numerical majority is in favour of the day very decidedly. Thus, in 56 cases, the attacks occurred nearly always in the day in 42, nearly always in the night in 11, and equally by day and night in 3. To complete this subject, though not strictly in place here, we may add, that epilepsy is essentially a chronic complaint, and may last any length of time within the ordinary limits of life, though, of course, with a tendency to shorten it; and that its attacks may occur at any intervals, from a few minutes, to months, or even years. There is occasionally, especially in old confirmed cases, a periodicity, but usually this is wanting, or extremely irregular. But this belongs to our next question.

V. *The Influences which preside over these and similar Phenomena.*— In answering this question, we are tempted to make very liberal extracts from Dr. Radcliffe's third chapter on Periodicity, as well to give an example of the pleasing style in which the work is written, as to afford us the required information. The illustrations used are the sensitive plant and the newt.

"The periodical changes in the life of the sensitive plant are both plain and simple. In spring the seedling emerges from the cradle in which it had slept during the winter; in summer it puts forth its foliage; in autumn it droops; in winter it dies. In spring it gives new signs of life; in summer it regains its verdure; in autumn it fades; and in winter it again becomes a bare and lifeless twig. Year by year these phenomena succeed each other with unfailing regularity, and the vitality ebbs and flows in direct relation to the ebbing and flowing intensity of the sunbeams.

"At daybreak also the leaves recover from the closed and pendant condition in which they have been all night, and—if not disturbed in any way—they remain erect and unfolded until evening, when they again close and droop; and these changes alternate with perfect regularity, so long as the leaves retain their characteristic irritability. In each case the vital movement corresponds with certain changes in the relative positions of the earth and sun; the one referring to the annual, the other to the diurnal revolution.

"The periodical changes in the life of the newt are not less plain and simple than those which occur in the life of the sensitive plant. The egg, like the seed, exhibits no sign of development, except it be quickened by the sunbeams, and the animal, like the plant, continues dependent upon the same fostering aid, throughout the whole course of its future life. As spring advances it grows day by day into a more active and sentient being; as autumn wanes it droops by degrees into a state of unbroken sleep. This winter slumber passes off at the renewal of spring, and returns at the end of autumn. . . . In the active period of its existence also the newt wakes in the day-time, and sleeps during the night. In a word, the life of this creature appears to be as closely wedded to the sun as that of the sensitive plant, and yet that life embraces a sentient principle, which is endowed with memory and other mysterious gifts.

"The diurnal changes in the life of the newt are reflected also by diurnal changes in the lives of other animals. Sleep still attends upon night, and wakefulness upon the day. At sunset the butterfly descends from the sky, the snail withdraws within her shell, the dace lies motionless in the pool, the frog ceases to leap across the path, the lark folds his wing and hushes his song, the deer retires to his lair, and sleep reigns over them during the night; but when the dawn illumines the east, the spell is broken, and all are released to life and enjoyment until the evening."

All this is no less philosophical in conception than beautiful in expression. In pursuing the subject the author shows how all vital activity is dependent upon, or closely related to, the amount of light and heat. He shows also, quoting Humboldt's eloquent account of the nocturnal life of animals, how the light of the moon has a similar influence to that of the sun, though in a less degree, and also that artificial light and heat have somewhat the same effects as the natural agents. It is then shown how, in the life and functions of man, there are distinct evidences of periodical action, and then we find this application of the doctrine to epilepsy:—

"It may be expected that the signs of periodicity will always be masked and obscure in man, but that they will be manifested most distinctly in him who is deprived of that active inherent life, which constitutes the badge of distinction between man and the plant, and not in the person who is acted upon by inflammation, or who is excited in any other way. And so it is.

"There can be no doubt as to the obscurity of the evidences of periodicity, even where that obscurity is least, as in epilepsy and the allied affections; but there can also be no doubt as to the existence of these evidences. Thus, on looking at a number of cases, it is found that convulsion and spasm occur more frequently at night than in the day; more frequently about the time of new moon than the time of full moon, and more frequently in the winter than in the summer months. Of these evidences of diurnal, monthly, and annual periodicity, the diurnal are the most frequent and the best established; but all are sufficiently frequent and obvious. And in this point of view the signs of periodicity become only so many additional evidences of that constitutional want of innate strength which appears to be the prominent fact in the pathology of epilepsy and the cognate disorders."—Epilepsy, p. 118—120.

It must, however, be acknowledged that as yet no general law of recurrence has been discovered to which epilepsy is amenable; and if the "formula of determination" be ever announced, it will of necessity contain so many "variable unknown quantities" as to render it nearly, if not altogether insusceptible of investigation in reference to individual instances. We have next to inquire—

VI. *What place in the natural history of disease does epilepsy claim?*—It is evident from what has been stated as to the morbid anatomy of this affection, that there are no changes sufficiently constant in the nervous centres to allow epilepsy a place in any anatomical classification of disease whatever. It is by its physiological relations that its true locality must be determined.

Epilepsy has generally been classed, apparently without doubt or misgiving, amongst the convulsive affections; yet, we think that a careful consideration of the phenomena will make its claim to this position appear less clear, notwithstanding that convulsion is so very frequent an attendant or *symptom*. We do not consider irritation of the neck of the bladder, or of the uterus, or dentition, or menstruation, as convulsive affections, on the grounds that convulsions frequently accompany these states. Passing slightly over the obvious difference between the *acute* nature of convulsions generally, and the *essentially chronic* nature of epilepsy, we have to notice the very important fact, that *spasmodic muscular action, though a frequent, is by no means a constant attendant upon epilepsy.* In the epileptic vertigo and many forms of the *petit mal* the convulsion is entirely or chiefly wanting, and in its place is a total and extreme relaxation of the whole muscular system. And these must not be considered as slight and imperfect attacks, for it is important to bear in mind that such patients as are affected with epileptic vertigo alone, are more rapidly and more constantly deteriorated in their intellectual functions than those in whom convulsion is prominent. It may be said that, even in these cases, there is some degree of convulsion, but surely so small an amount of any action as that which is imperceptible can scarcely be sufficient to characterize a disease. We saw very recently an epileptic attack which lasted above twenty-four hours, where the whole muscular system was in a state of the most complete relaxation, and the most careful investigation failed to discover any indications of spasm. That these and similar cases are *truly* epileptic, the history, connexions, and general symptoms sufficiently prove. If this be so, we conceive that epilepsy has no claim to be considered essentially a convulsive affection. The one constant symptom is, loss (or great diminution or embarrassment) of consciousness, accompanied with considerable *modification* of the muscular system.

What, then, is the position of this disease nosologically? We pass over all those opinions as untenable, which connect it with inflammation of the white matter of the brain, with alterations in the pineal body, or with any constant change whatever. Dr. Cheyne writes thus:—

"We conceive that epilepsy is as certain a manifestation of the strumous diathesis as tubercular consumption, psoas abscess, hereditary insanity, or certain congenital malformations or defects of organization, which are inherited only from scrofulous parents. We have no recollection of a case of cerebral epilepsy in a patient, who, when due inquiry was made, did not appear to inherit a strong disposition to scrofula."—"Cyclopædia of Practical Medicine," article—Epilepsy.

This appears a very probable hypothesis, but by way of further indicating the connexions of epilepsy, we will refer once more to its ultimate phenomena. A person, apparently in good health, is seized with an epileptic fit; in a few hours or a few days at most he is in perfect health again. After an interval more or less prolonged the attack returns, and again and again departs, leaving no particular alteration behind in any of the functions. But, by degrees more or less insidious, a change is observed, perhaps first in the memory, perhaps in the motor functions, gradually augmenting till it terminates in mental alienation and paralysis, perfect or imperfect, and, finally, in death. Mental alienation, as a result of epilepsy, is so frequent, as almost to be considered a constant termination of those cases which last long enough.

Esquirol found, amongst 339 epileptics, 269 in a state of mental alienation, a very large proportion, and one which would be increased if the final history of the remainder could have been investigated. In such cases as these, then, the final condition is one of mental deterioration, muscular degeneration, and occasional convulsive attacks. The morbid appearances usually found are, adhesions of the membranes, sometimes with thickening and opacity, induration of the white matter (but *occasional* softening); the same changes in the grey matter with a mottled appearance. (M. Foville.) These appearances are precisely identical with those found in another class of cases, viz., insanity complicated with paralysis. The history of these is similar to that of the others, with this exception, that in these the psychical degeneration comes on first, and is succeeded by the muscular degradation, and, finally, by the epileptiform seizures which are so constant an attendant upon this form of insanity; the final condition is the same—mental deterioration, muscular degeneration, and occasional convulsive attacks. This similarity of history with *identity* of results, whether we regard the last living state or the morbid appearances after death, cannot fail to indicate strongly and clearly the close connexion which exists between the two diseases; and we therefore conclude that epilepsy is much more closely allied to insanity than to convulsive affections in general. The most frequent form under which insanity invades the epileptic patient is *dementia*, the next, mania; monomania is occasional, but very rare. (Esquirol.) We need scarcely add our testimony to the almost universal conviction of the intractable nature of these *allied* affections; *singly* they are frequently amenable to treatment, but, whether commencing by epilepsy and passing into insanity, or by insanity passing into epileptiform attacks, no sooner does the one threaten to complicate the other than the prognosis is much more unfavourable, and almost hopeless.

*To be continued.*

